# Disruption of the *Arabidopsis* Acyl-Activating Enzyme 3 Impairs Seed Coat Mucilage Accumulation and Seed Germination

**DOI:** 10.3390/ijms25021149

**Published:** 2024-01-17

**Authors:** Ninghui Cheng, Paul A. Nakata

**Affiliations:** USDA/ARS Children’s Nutrition Research Center, Department of Pediatrics, Baylor College of Medicine, Houston, TX 77030-2600, USA; ncheng@bcm.edu

**Keywords:** *Arabidopsis*, acyl-activating enzyme, seed mucilage, oxalate

## Abstract

The Acyl-activating enzyme (*AAE*) 3 gene encodes an oxalyl-CoA synthetase that catalyzes the conversion of oxalate to oxalyl-CoA as the first step in the CoA-dependent pathway of oxalate catabolism. Although the role of this enzyme in oxalate catabolism has been established, its biological roles in plant growth and development are less understood. As a step toward gaining a better understanding of these biological roles, we report here a characterization of the *Arabidopsis* thaliana *aae3* (*Ataae3*) seed mucilage phenotype. Ruthidium red (RR) staining of *Ataae3* and wild type (WT) seeds suggested that the observed reduction in *Ataae3* germination may be attributable, at least in part, to a decrease in seed mucilage accumulation. Quantitative RT-PCR analysis revealed that the expression of selected mucilage regulatory transcription factors, as well as of biosynthetic and extrusion genes, was significantly down-regulated in the *Ataae3* seeds. Mucilage accumulation in seeds from an engineered oxalate-accumulating *Arabidopsis* and *Atoxc* mutant, blocked in the second step of the CoA-dependent pathway of oxalate catabolism, were found to be similar to WT. These findings suggest that elevated tissue oxalate concentrations and loss of the oxalate catabolism pathway downstream of AAE3 were not responsible for the reduced *Ataae3* seed germination and mucilage phenotypes. Overall, our findings unveil the presence of regulatory interplay between AAE3 and transcriptional control of mucilage gene expression.

## 1. Introduction

Seed coat mucilage, known as myxospermy, is present in more than 80 families of angiosperms, including the model plant *Arabidopsis thaliana* [1]. Seed coat mucilage is produced by the mucilage secretory cells (MSCs) which make up the outermost layer of the seed coat. Once produced, the mucilage is extruded into the apoplast—the space between the primary cell wall and plasma membrane—of the seed coat [2,3]. Seed coat mucilage is composed of pectin, cellulose, hemicellulose, and protein [4,5,6,7]. The cellulose plays a critical structural role, while the pectic polysaccharides account for the hydrophilic nature of mucilage [7,8].

This hydrophilic property allows the mucilage, upon exposure to an aqueous environment, to encapsulate the seed in a gel-like transparent capsule [1,4,9]. The moist environment provided by the hydrated mucilage is important in facilitating seed imbibition, metabolic activity, and, ultimately, germination. Other reported functions for seed mucilage include roles in seed dispersal [1,9,10,11] and as a signaling molecule in interactions between plants and pathogens [12,13].

The production of seed coat mucilage is a highly regulated process [2,14,15,16]. Genetic analysis of seed coat development in *Arabidopsis* identified an array of genetic loci and/or genes that are involved in MSC differentiation, mucilage biosynthesis, pectin modification, and mucilage extrusion [1,11,14,15]. Recent advances in seed biology have also provided insight into the function of specific transcription factors (TFs) in regulating seed coat development and mucilage production [17]. In addition, proteomic analysis has revealed that *Arabidopsis* seed coat mucilage contains some mucilage-specific proteins, suggesting that a unique set of genes involved in mucilage accumulation are expressed during seed coat development [5].

Even though the mucilage is just present in the seed coat, the appropriate levels of metabolic and photosynthetic activity are evident in developing seeds, suggesting that these processes may play a critical role in seed coat development and mucilage formation [18]. Oxalic acid (oxalate), an end product of carbon metabolism, has been shown to play a critical role in many biological and metabolic processes in plants [19]. In soybean, oxalate has been shown to accumulate in the seed coat in the form of the calcium oxalate crystal during seed coat development and embryo maturation [20]. However, metabolic (oxalate) regulation and contribution to seed coat development, as well as mucilage production and maturation, are poorly understood.

Phenotypic analysis of an *Ataae3* T-DNA mutant revealed that a lack of AAE3, an *acyl-activating enzyme 3* [21], resulted in an increase in oxalate accumulation, a reduction in vegetative growth, a reduction in seed mucilage production, and a reduction in seed germination [22]. Characterization of the *Medicago truncatula aae3* (*Mtaae3*) RNAi knock-down and *Mtaae3 Tnt1* knock-out mutants [23] also revealed an increase in oxalate accumulation, a reduction in vegetative growth, a delay in seed germination, and a defect in seed coat development [24]. AAE3 has been shown to encode an oxalyl CoA synthetase that catalyzes the first step in a previously uncharacterized pathway of oxalate catabolism in plants and yeast [22,23,24,25,26,27,28,29]. Although the biochemical activity of AAE3 has been established, its function in plant growth and development remains unclear.

In the present study, we expand our investigation into the role of AAE3 in seed coat mucilage formation and extrusion. Quantitative RT-PCR was employed to analyze the expression of selected genes involved in seed coat mucilage biosynthesis and secretion. Seeds from oxalate-accumulating *Arabidopsis* transgenic plants (*Obc1* plants) were assayed for mucilage accumulation, mucilage gene expression, and seed germination to assess whether the observed *Ataae3* germination and mucilage phenotypes were related to the elevated tissue oxalate concentrations that resulted from the inability of *Ataae3* to catabolize oxalate. Similarly, seeds from an *Arabidopsis* mutant, *Atoxc,* that lack the oxalyl-CoA decarboxylase required to catalyze the second step in the CoA-dependent pathway of oxalate catabolism, were evaluated for the accumulation of mucilage to determine whether *Ataae3*′s reduced mucilage phenotype was caused by the absence of a downstream pathway metabolite. Overall, our findings show that *At*AAE3’s functional role extends beyond oxalate catabolism into other processes such as seed mucilage accumulation and germination.

## 2. Results and Discussion

### 2.1. Disruption of AAE3 Reduces Seed Germination and Mucilage Accumulation

**Figure 1:**
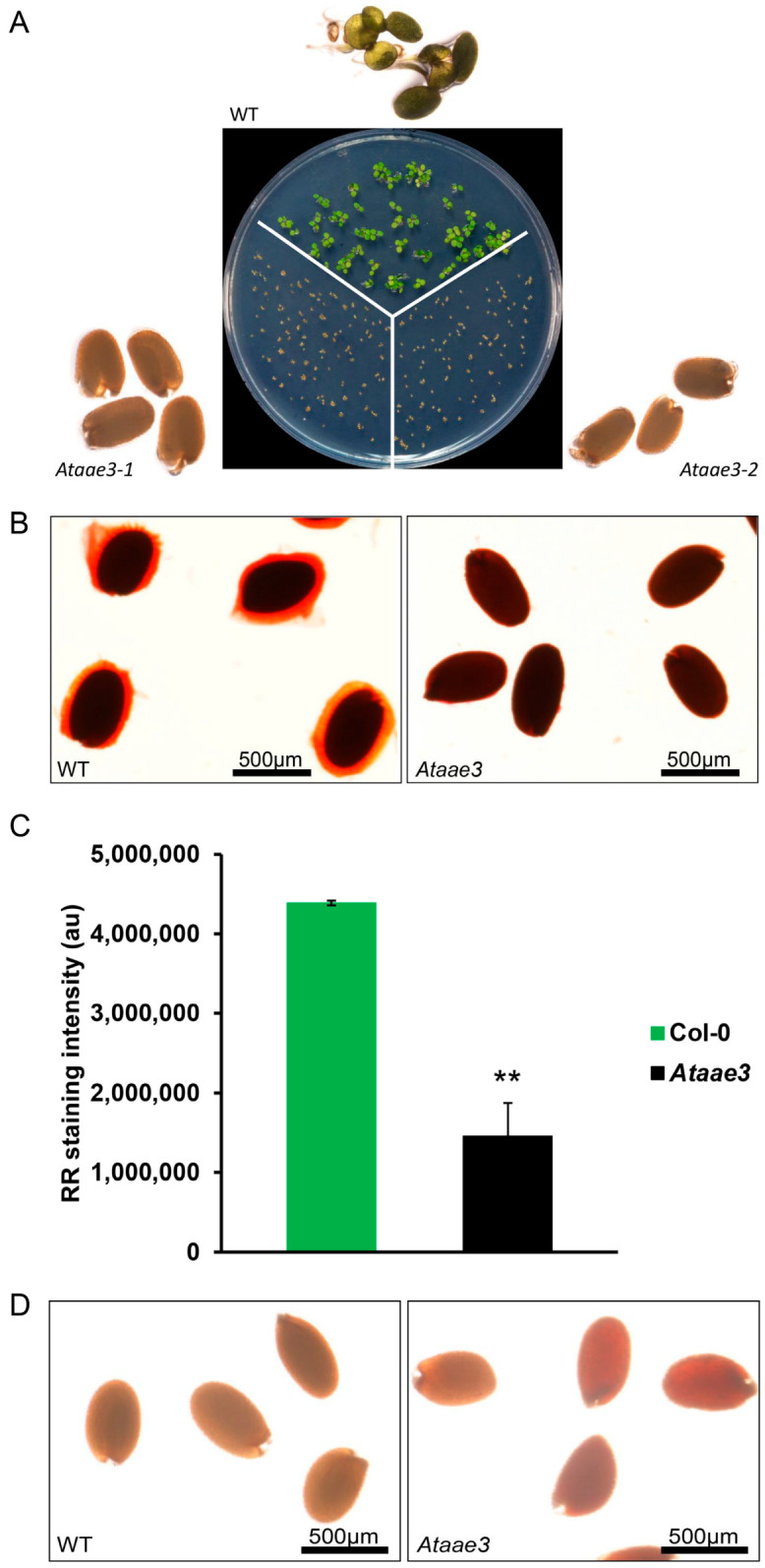
Deletion of AtAAE3 impairs seed germination, seed coat mucilage accumulation, and increases seed permeability. (**A**) *Arabidopsis* WT and *Ataae3* seeds were germinated on 0.5× MS media with 0.5% sucrose for 4 days. (**B**) *Arabidopsis* WT and *Ataae3* seeds were stained with ruthenium red solution to visualize mucilage accumulation on the seed coats. (**C**) Quantification of mucilage in *Ataae3* and WT seeds. RR staining intensity was quantified using Image J (ij153-win-java8). Student’s *t*-test; *n* = 3; ** *p* < 0.01 indicates a statistically significant difference between mutant seeds and wild type controls. (**D**) Seeds were stained with 2,3,5-triphenyltetrazolium chloride to evaluate seed coat permeability. Scale bars = 500 µm.

Although *Ataae*3 seeds looked indistinguishable from WT controls (Supplementary Figure S1), seed germination assays showed a significant delay in *Ataae3* seed germination compared to WT (Figure 1A). Ruthenium red (RR) staining of WT and *Ataae3* seeds revealed that the *Ataae3* seeds lacked the mucilage coating that encompassed the WT seeds (Figure 1B). To assess whether *Ataae3* seeds had an alteration in the biosynthesis and/or extrusion of mucilage, water-soluble extracts of *Ataae3* and WT seeds were semi-quantified for mucilage content using a RR dot-blot staining assay. As shown in Figure 1C, *Ataae3* seeds still possessed the ability to produce some mucilage, but this mucilage appeared to be retained within the seed coat. Thus, *Ataae3* seeds displayed a reduction in mucilage biosynthesis and extrusion. In addition, staining with 2,3,5-triphenyltetrazolium chloride revealed that *Ataae3* seeds had higher seed coat permeability in comparison with WT controls (Figure 1D) [22]. Thus, the lack of AAE3 activity also affected other aspects of seed coat development. Seed germinations are complicated biological processes that are controlled by many factors [30,31]. It has been known that seed mucilage is crucial for controlling seed dormancy and germination [32,33,34]. Therefore, our findings indicated that the observed reduction in seed mucilage accumulation may explain, at least in part, the observed reduction in *Ataae3* seed germination.

### 2.2. Disruption of AAE3 Reduces Mucilage-Related Gene Expression

**Figure 2:**
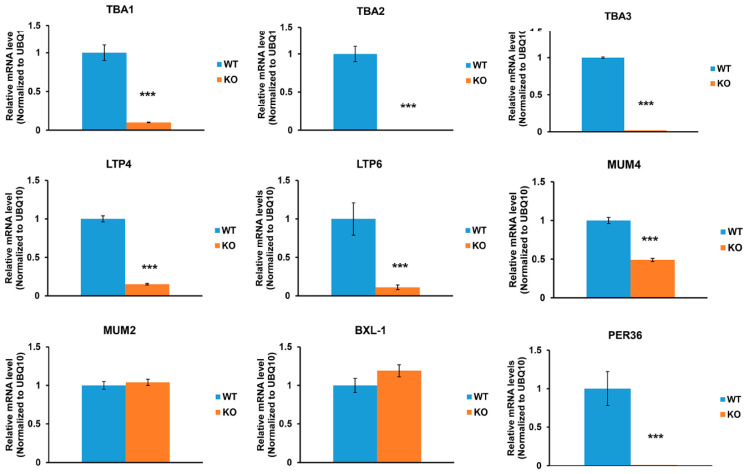
Effect of *AtAAE3* on mucilage-related gene expression. Disruption of *AtAAE3* significantly suppresses seed coat mucilage gene expression, as revealed by q-RT-PCR. Student’s *t*-test; *n* = 3; *** *p* < 0.001 indicates a significant difference between mutant seeds and wild type controls.

As a step toward uncovering the connection between the observed reduction in seed mucilage accumulation and a lack of *At*AAE3 activity, we assessed the expression of different mucilage-related genes using q-RT-PCR. The first set of genes we analyzed were a group of abundant seed mucilage proteins, called testa abundant proteins (TBA1 through 3), and two lipid transfer proteins (LTP 4 and 6). These proteins were identified as seed mucilage proteins by proteomic analysis [5]. The expression of the three *TBA* and two *LTP* genes were found to be significantly reduced in developing *Ataae3* seeds compared to WT controls (Figure 2).

Next, we measured the expression of several key genes, shown by genetic studies [14] to be involved in seed coat mucilage biosynthesis and extrusion. Expression of mucilage-modified 4 (*MUM4*), encoding a putative NDP-L-rhamnose synthase that is required for the synthesis of the pectin rhamnogalacturonan I [35,36], was found to be significantly reduced in *Ataae3* seeds compared to WT (Figure 2). *MUM2,* encoding a *β*-Galactosidase that is required for maturation of rhamnogalacturonan I [37,38], and *AtBXL1,* encoding a bifunctional *β*-D-xylosidase/*α*-L-arabinofuranosidase that is involved in pectic arabinan modification [39], play critical roles in seed mucilage release. Q-PCR analysis indicated that the expression of *MUM2* and *AtBXL1* was not changed in *Ataae3* seeds compared to WT controls (Figure 2). By contrast, the expression of a gene encoding a class III peroxidase, peroxidase 36 (PER36), was significantly decreased in *Ataae3* seeds (Figure 2). *PER36* is expressed exclusively in the outer integument (oi2) cell and is required for mucilage extrusion [40]. Our findings indicate that *PER36* may contribute to the lack of mucilage extrusion in *Ataee3* seeds. Overall, these results support a link between the expression of *AtAAE3* and seed mucilage extrusion.

### 2.3. Disruption of AAE3 Reduces Mucilage Regulatory Gene Expression

**Figure 3:**
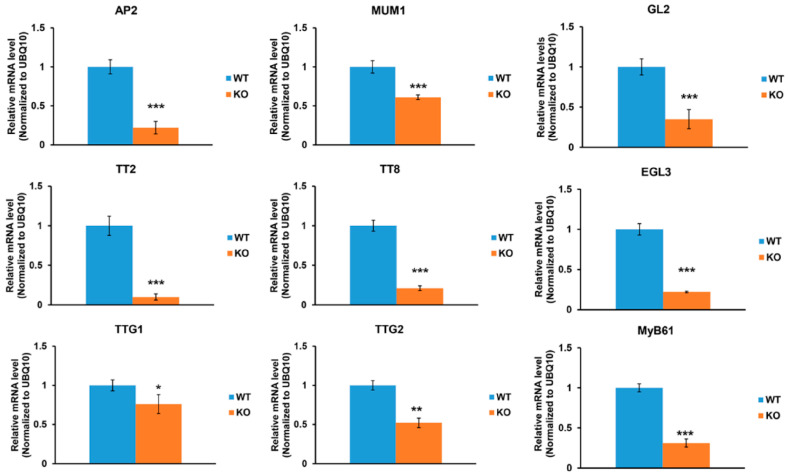
Effect of *AtAAE3* on mucilage-related transcription factor expression. Disruption of *AtAAE3* significantly suppresses expression of regulatory transcription factor genes involved in seed mucilage formation and seed coat development. Student’s *t*-test; *n* = 3; * *p* < 0.05, ** *p* < 0.01, and *** *p* < 0.001 indicate a significant difference between mutant seeds and wild type controls.

Q-RT-PCR analysis of a group of key TFs [17] that regulate seed mucilage accumulation showed that the expression of each tested *TF* was down-regulated in *Ataae3* seeds compared to WT controls (Figure 3). The floral homeotic *TF* gene, *APETALA2* (*AP2*), has been shown to regulate seed coat mucilage biosynthesis [14] in addition to flower and ovule development [41]. The measured reduction in *AP2* expression in *Ataee3* seeds correlated with the decreased mucilage production in the mutant seed (Figure 1C). The MYB (v-Myb myeloblastosis viral oncogene homolog)–bHLH (basic helix–loop–helix)–WD40 domain (MBW) TF complex, consisting of TRANSPARENT TESTA GLABRA1 (TTG1), MYBs, TRANSPARENT TESTA2 (TT2), TT8, and ENHANCER OF GLABRA3 (EGL3) [42,43,44], controls seed coat differentiation and mucilage through regulation of *TTG2* and *GL2* [17]. The expression of *TTG1*, *TT2*, *TT8*, and *EGL3*, and of their target genes, *TTG2* and *GL2*, were significantly down-regulated in *Ataae3* seeds compared to WT controls (Figure 3). The MYB–bHLH–WD40 (MBW) TF complex is thought to function in seed coat differentiation and mucilage deposition in a manner that is independent of the AP2 regulatory pathway [17].

*MYB61*, a member of the R2R3-MYB transcription factor family, has been shown to be critical for seed coat mucilage deposition and extrusion [45]. MYB61 is thought to function in a genetic pathway distinct from that of TTG1 in seed coat differentiation and development [45]. In *Ataae3* seeds, the expression of *MYB61*, like *TTG1*, was significantly reduced compared to WT controls (Figure 3). It has been shown that the transcription factor, *MUM1/LUH*, is independent of other TFs in seed mucilage release as a result of positive regulation of *MUM2* and *AtBXL1* [46]. The expression of *MUM1* in *Ataae3* seeds was significantly reduced in comparison to WT controls (Figure 3). Interestingly, both *MUM2* and *AtBXL1* expressions, as distinct from *MUM1*, were not changed (Figure 2). It is possible that *MUM1* controls *MUM2* and *AtBXL1* expression indirectly, through an unidentified regulator [47] whose expression could also be altered in *Ataae3* seeds. Overall, the determined decrease in the expression of key seed mucilage regulatory genes in *Ataae3* seeds is consistent with the measured decrease in *Ataae3* seed mucilage accumulation.

### 2.4. An Increase in Oxalate Accumulation Affects Seed Coat Development, but Not Mucilage Accumulation

**Figure 4:**
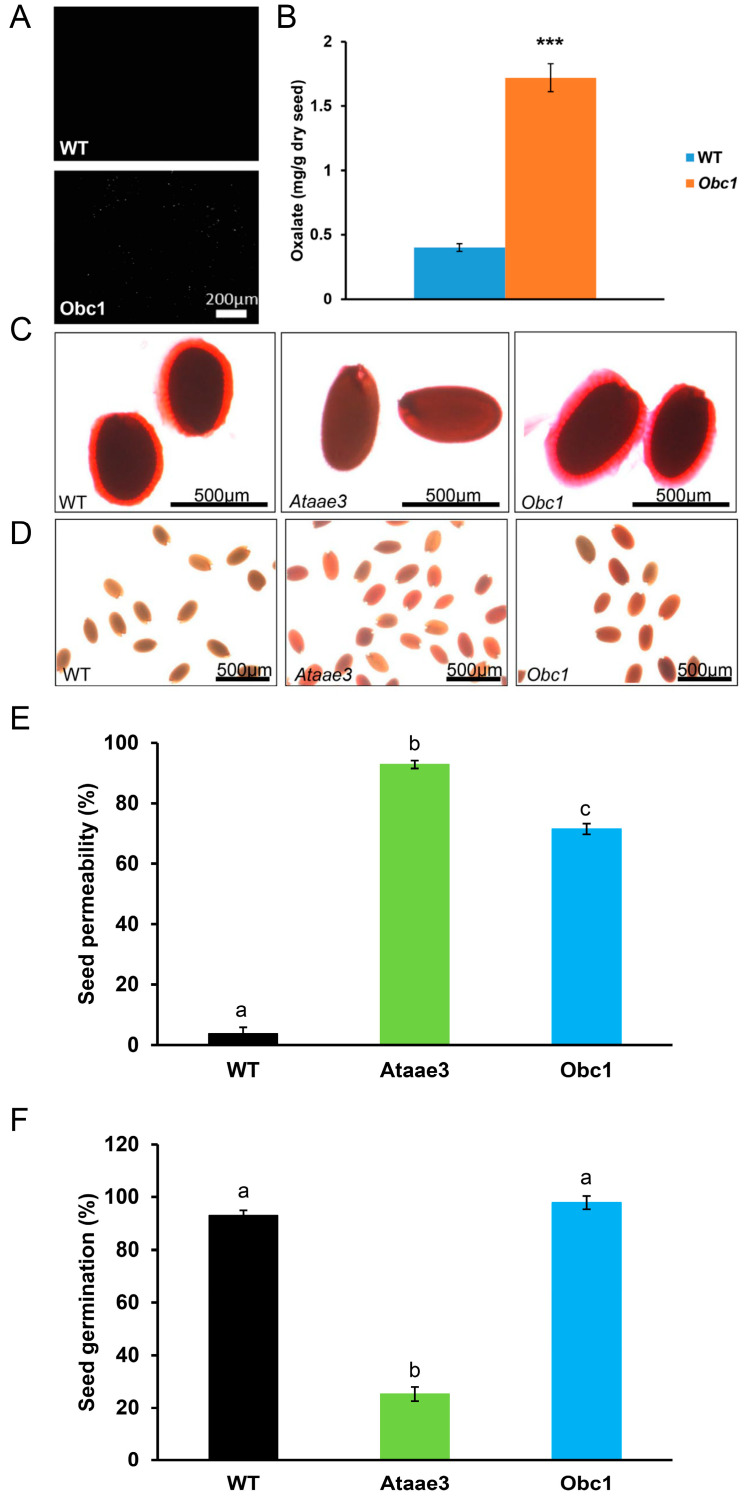
Effect of excess oxalate on seed coat mucilage and cell wall development. Overexpression of *Obc1* in *Arabidopsis* increases calcium oxalate crystal accumulation in (**A**) leaf, and (**B**) seeds. Student’s *t*-test; *n* = 3; *** *p* < 0.001 indicates a significant difference between *Obc1* seeds and wild type controls. (**C**) *Arabidopsis* WT and *Obc1* seeds were stained with RR to visualize mucilage accumulation on the seed coats. (**D**) Seeds were stained with 2,3,5-triphenyltetrazolium chloride to evaluate the seed coat permeability. Scale bars = 500 µm. (**E**) Quantification of seed permeability (%). *n* > 100; a, b, and c indicate significant differences between wild type, *Ataae3*, and *Obc1* seeds. Accumulation of oxalate content in *Obc1* seeds does not change seed mucilage formation but significantly alters seed coat permeability. (**F**) *Arabidopsis* WT, *Ataae3*, and *Obc1* seeds were germinated on a 0.5× MS medium, using 0.5% sucrose, for 4 days. Seed germination (%) was determined. *n* > 100; a and b indicate significant differences between WT, *Ataae3*, and *Obc1* seeds. There was no difference in seed germination between WT and *Obc1* seeds.

**Figure 5:**
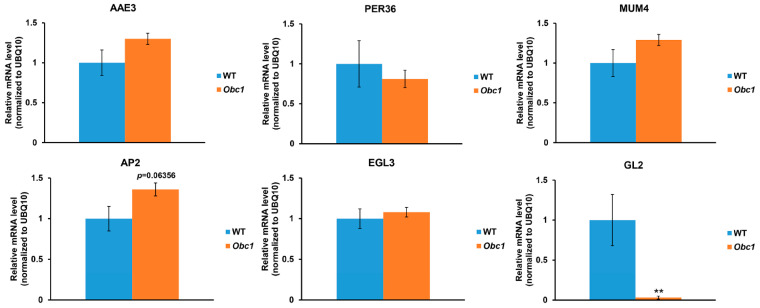
Effect of excess oxalate on mucilage gene expression. Q-RT-PCR analysis of RNA samples extracted from wild type and *Obc1* siliques. The results indicate that accumulation of oxalate content does not change the expression of seed mucilage genes but does affect the expression of some regulatory genes involved in seed coat development. Student’s *t*-test; *n* = 3; ** *p* < 0.01 indicates a significant difference between wild type and *Obc1* seeds.

Loss of *AtAAE3* in *Arabidopsis* blocks the CoA-dependent pathway of oxalate catabolism and increases the accumulation of oxalate in the leaf tissues (WT: 0.75 ± 0.2 mg/g dry wt vs. *Ataae3*: 1.3 ± 0.3 mg/g dry wt [22]) as well as in mature seeds (WT: 1.8 ± 0.2 mg/g dry wt vs. *Ataae3*: 6.0 ± 1.0 mg/g dry wt [22]). It has been proposed that the increased accumulation of tissue oxalate could result in the disruption of seed coat mucilage production and cell wall development [22]. To test this proposal, we utilized an *Arabidopsis* transgenic line [48] expressing the oxalate biosynthetic component 1 (*Obc1*) gene, cloned from the animal bacterial pathogen *Burkholderia mallei* [49]. The *Obc1* transgenic plants accumulated higher tissue oxalate concentrations and formed crystals (Figure 4A,B) of calcium oxalate [48]. Although *Obc1* seeds had higher oxalate concentrations than controls (Figure 4B), RR staining revealed that these seeds accumulated mucilage in a manner that was similar to WT controls (Figure 4C). Likewise, qRT-PCR analysis showed that the expression of mucilage-related genes was not changed in *Obc1* seeds compared to WT controls (Figure 5). Taken together, our finding suggests that the accumulation of oxalate in the *Ataae3* seeds is not the cause of the reduction in mucilage accumulation.

In contrast to mucilage accumulation, *Obc1* seeds showed a significant increase in seed coat permeability in comparison to WT controls (Figure 4D,E), suggesting that excess oxalate may affect seed cell wall development. As found in *Ataae3* seeds (Figure 3), *GL2* expression was also significantly reduced in *Obc1* seeds compared to WT controls (Figure 5). It is possible that this alteration in expression may be linked to the change in seed coat permeability observed in *Ataae3* and *Obc1* (Figure 4D,E). Further studies, however, are required before any firm conclusions can be drawn.

To assess the effect of elevated tissue oxalate concentration on seed germination, WT, *Ataae3*, and *Obc1* seeds were plated as previously described [22]. In contrast to *Ataae3*, *Obc1* seeds were found to germinate in a similar manner to WT controls (Figure 4F). This finding suggested that the reduction in *Ataae3* seed germination was attributable to a lack of mucilage rather than to an alteration in seed permeability.

### 2.5. AAE3 Is Critical for Seed Mucilage Accumulation

**Figure 6:**
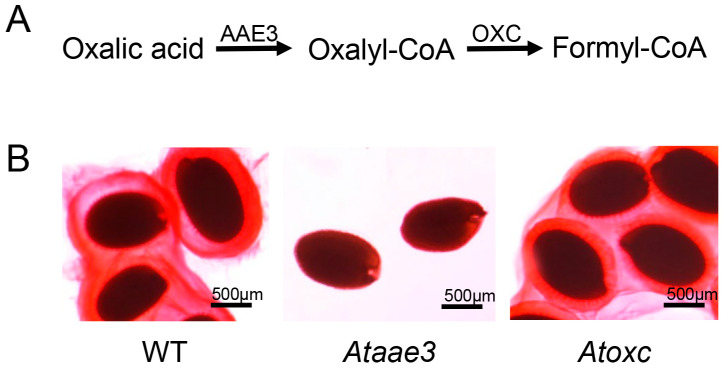
*At*AAE3 is critical for mucilage accumulation. (**A**) First two steps in the CoA-dependent pathway of oxalate catabolism. (**B**) *Arabidopsis* WT, *Ataae3*, and *Atoxc* seeds were stained with RR to visualize mucilage accumulation on the seed coats. The results show that the reduction in mucilage accumulation is specific to the *Ataae3* and does not result from a lack of a particular downstream metabolic intermediate due to blockage of the CoA-dependent pathway of oxalate catabolism.

To determine whether the lack of mucilage was specific to the loss of AAE3 function and not a general phenomenon resulting from a missing downstream metabolite due to disruption of the CoA-dependent pathway of oxalate catabolism (Figure 6A), seeds from the *Atoxc* mutant were stained to reveal the presence of mucilage (Figure 6B). The *At*OXC has been shown to catalyze the second step in the CoA-dependent pathway of oxalate catabolism [50,51]. Loss of *At*OXC in *Arabidopsis* reduces the degradation of oxalate and increases the accumulation of seed oxalate concentration (WT: 0.7 ± 0.1 mg/g dry wt vs. *Atoxc*: 2.7 ± 0.3 mg/g dry wt [51]). RR staining revealed that *Atoxc* seeds accumulate ample amounts of mucilage, similar to WT (Figure 6B). This finding shows that the reduction in mucilage is specific to the *Ataae3* mutant and does not result from a missing downstream metabolite from the CoA pathway of oxalate catabolism.

Our findings demonstrate that *At*AAE3 is crucial for seed cell wall development and for mucilage production and extrusion (Figure 1, Figure 4 and Figure 6). *At*AAE3 belongs to the acyl-activating enzyme superfamily that plays an important role in the biosynthesis of many metabolites [52]. For instance, long-chain acyl-CoA synthetase (LACS) 1 and 2 in the clade I of the *AAE* superfamily are required for biosynthesis of cuticular wax products, and deletion of *LACS* 1 and *2* genes results in organ fusion, defective flower development, and reduced seed production [53]. Like *AAE3*, *AAE13* is in clade VII of the *AAE* superfamily and encodes a malonyl-CoA synthetase that is critical for anthocyanin biosynthesis, plant growth, and seed set production [54,55]. Furthermore, *Arabidopsis AAE9*, encoding an isobutyl-CoA synthetase, has been shown to be a key factor involved in branched-chain amino acid catabolism in *iso*-branched wax biosynthesis [56]. It was recently reported that disruption of *SlAAE3* expression in tomato significantly reduced the degradation of tissue oxalate and affected tomato fruit quality through an alteration in nutrient metabolites concentrations [29]. TmAAE3 from Taxus × media has been shown to be involved in the activation of 4-methylbutyric acid (*N*-debenzoyl-*N*-(2-methylbutyryl) taxol side chain) biosynthesis [57]. Interestingly, a recent report also demonstrates that LsAAE3 from grass pea is part of another biosynthetic pathway in that it provides the substrate for the production of the neurotoxin, β-L-oxalyl-2,3-diaminopropionic acid (β-L-ODAP) [58]. These studies suggest diverse roles for AAE family members, including AAE3, in metabolite biosynthesis, plant growth, and organ development. Nevertheless, the mechanisms by which *At*AAE3 affects seed coat mucilage formation and extrusion remains unknown and will be the focus of future investigations.

**Figure 7:**
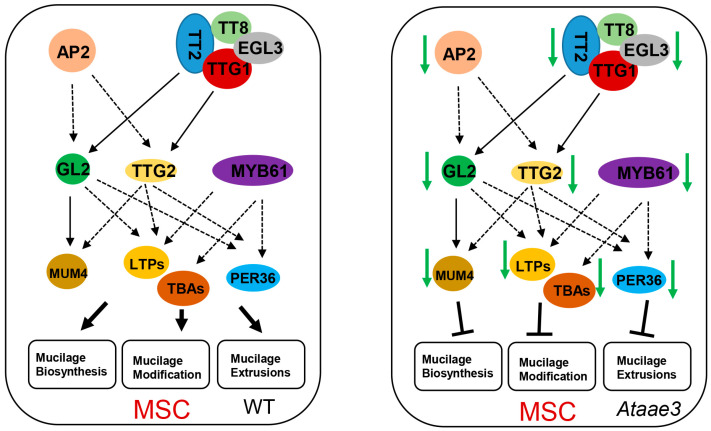
A working hypothesis for the role of AtAAE3 in seed mucilage formation and seed coat development. Shown in the left panel is the expression of the TF regulatory network and the genes involved in mucilage production and extrusion in WT mucilage secretory cells (MSCs). In the *Ataae3* mucilage secretory cells (MSCs) (right panel), expression of the TFs and their target genes is significantly down-regulated, reducing mucilage production and mucilage extrusion.

In conclusion, we showed that a loss of *AAE3* function results in an increase in tissue oxalate concentrations, decreased seed germination, and reduced seed coat mucilage accumulation. Disruption of *AAE3* significantly down-regulates the expression of key mucilage-biosynthetic and extrusion-related genes, as well as of major regulatory transcription factors in developing seeds that contribute to the decrease in mucilage formation and extrusion, as well as to the defect in seed coat permeability (Figure 7). Our findings indicate that AAE3 plays a critical role in seed cell wall development, and that it offers a clue as to the potential regulatory interplay between metabolite homeostasis and gene transcriptional control in mucilage production and extrusion.

## 3. Materials and Methods

### 3.1. Reagents

All chemicals were purchased from Sigma-Aldrich (St. Louis, MO, USA) unless stated otherwise. The oxalate diagnostic kit was purchased from Trinity Biotech (Jamestown, NY, USA).

### 3.2. Plant Materials and Growth Conditions

Wild type (WT, ecotype Columbia, Col-0), *Ataae3* T-DNA insertional line, *Obc1* over-expression lines, and *Atoxc* mutant lines were described previously [22,48,51]. For *Arabidopsis* growth, seeds were sown on commercial soil (Pro-Line, growing mix, C/20, Jolly Gardener, Oldcastle Lawn and Garden, Inc., Poland Spring, ME, USA), and grown under 100–200 μE using a 16 h day/8 h night photoperiod at 22 °C.

### 3.3. Dry Seed Mucilage Extraction and Measurement

The extraction of seed mucilage was conducted following a modified procedure from a previous report [45]. In brief, dry WT and *Ataae3* seeds were ground to a fine powder using a mortar and pestle. Equal amounts of dry seed powder were homogenized in 2 M imidazole (pH 7.5) and centrifuged to remove the insoluble matter. The recovered supernatant was extracted with five volumes of ethanol and centrifuged. After removal of the supernatant, the precipitated material was dissolved in water. This precipitation step was repeated three times.

After the final resolubilization step, the soluble polysaccharides were quantified by dot-blot assay, following a published protocol [59]. In brief, 5 µL of the extracted samples were applied to a positively charged nylon membrane. The sample material was fixed to the membrane by heating at 70 °C for 5 min, followed by two brief washes in water to remove excess salts. The membranes were then stained with ruthenium red (RR) solution (34 µM) at room temperature (RT) for 15 min. After staining, the membranes were rinsed with water and dried at RT. Each membrane was scanned, and the RR staining intensities were quantified using Image J (ij153-win-java8).

### 3.4. Analysis of Seed Phenotypes

To visualize seed mucilage, *Arabidopsis* WT, *Ataae3*, *Obc1,* and *Atoxc* seeds were stained with RR. In brief, the seeds were incubated for 15 min in a 0.03% (*w*/*v*) RR solution, rinsed gently with water, and viewed using a light microscope as previously described [22]. To determine seed permeability, *Arabidopsis* WT, *Ataae3*, and *Obc1* seeds were stained with 1% (*w*/*v*) 2,3,5-triphenyltetrazolium chloride at 30 °C for 24 h, as previously described [22]. After staining, seeds were rinsed with water and viewed under the light microscope.

### 3.5. Microscopic Analysis of Calcium Oxalate Crystal in Arabidopsis Leaves

Leaf samples were harvested from WT and *Obc1* plants and cleared in 95% (*v*/*v*) ethanol. The leaf samples were then equilibrated with water and visually inspected for calcium oxalate crystal deposition using light microscopy and crossed-polarizers. Images of whole-leaf mounts were captured using a CCD72 camera mounted on a Zeiss Axiophot light microscope [48].

### 3.6. Oxalate Measurements

Oxalate determinations were carried out as previously described [48], with some modifications. Dried WT and *Obc1* mutant seed samples were weighed and ground in water using a mortar and pestle. Total oxalate concentrations were determined by simply solubilizing the crystals with the addition of H^+^-Dowex in 4 mM sulfuric acid. The samples were incubated at 60 °C for 1 h to dissolve the oxalate crystals. The pH of the mixture was then adjusted (pH 5–7), followed by charcoal filtration and centrifugation. The supernatant was then analyzed for oxalate content according to the manufacturer’s instructions. Standards were prepared from oxalic acid dihydrate and used for both soluble and total oxalate measurements, as recommended by the manufacturer. Measurements were performed in duplicate on three independently grown sets of plants, the results averaged, and standard errors calculated [48].

### 3.7. RNA Isolation, cDNA Synthesis, and qRT-PCR Analysis

Gene expression analysis was conducted following a previously published procedure [60]. Siliques (7 dap) were collected from WT, *Ataae3*, and *Obc1* plants, and analyzed for mucilage gene expression following the guidance provided in previous reports [2,3,61]. In brief, 100 mg of siliques collected from each plant line were individually pooled. Total RNA was extracted from each silique pool using the RNAqueous Kit (Ambion, Austin, TX, USA) following the instruction provided by the manufacturer. Isolated total RNA samples were further purified through treatment with DNase I (RNase-free, New England Biolabs, Ipswich, MA, USA). First-strand cDNA was synthesized using random hexamers and reverse transcriptase (ThermoFisher Scientific, Waltham, MA, USA). The resulting cDNA was diluted to 250 ng/µL, and 2 µL of the diluted cDNA was used as a template for each qRT-PCR reaction. qRT-PCR was performed using the SYBR green-based system and the Bio-Rad CFX96™. CFX Maestro software version 2.3 was used for data collection and analysis. Relative mRNA levels were normalized to an internal reference. Primers used are listed in Supplementary Table S1.

### 3.8. Statistical Analysis

All results are reported as means ± SEM. An analysis of variance was used to analyze data. Student’s *t*-test was used to compare the two groups. * *p* < 0.05, ** *p* < 0.01, and *** *p* < 0.001 were used as indicators of the level of statistical significance.

## Data Availability

Data are contained within the article or Supplementary Materials.

## Supplementary Materials

The following supporting information can be downloaded at https://www.mdpi.com/article/10.3390/ijms25021149/s1.
