# Supplementary material for: Disruption of the Arabidopsis Acyl-Activating Enzyme 3 Impairs Seed Coat Mucilage Accumulation and Seed Germination
Source: Int J Mol Sci. 2024 Jan 17;25(2):1149. doi: 10.3390/ijms25021149 (PMC10816874; doi:10.3390/ijms25021149)
Supplement: Supplementary file 1 [file ijms-25-01149-s001.zip › ijms-2791198-supplementary.pdf]

Supplementary Table S1. Primers used for q-RT-PCR analysis.

AtAAE3 forward	GTTGCATTCGGTGTTCCTG
AtAAE3 reverse	TTGGAATCACCGCACAGTTA
UBQ10 forward	GCTTCGTTTTTATTATCTGTGCTTCTT
UBQ10 reverse	TCGCAGAACTGCACTAAACAGAGT
AtTBA1 forward	AACTCACCACAAACACAATCTAATC
AtTBA1 reverse	TTGGTCTCCGTGGACAAATC
AtTBA2 forward	GAGACCAAATTAGGCACCTCTC
AtTBA2 reverse	CCTGGACCTTTGGGATTGTT
AtTBA3 forward	ACGTGCAAAGGGTAAGGTAG
AtTBA3 reverse	GAGATGACCTCACGCTCAAT
AtMUM2 forward	GCTTCTCAAGGAGGTCCAATTA
AtMUM2 reverse	TGATGTACGATGCTCCTTTCTC
AtBXL1 forward	GTACCGATCCATGTGAGAGTTC
AtBXL1 reverse	CACCACTCATAGCCTCCAATAC
AtMUM4 forward	CTGTTACCGACGAATCCTTACTC
AtMUM4 reverse	GTTCCCGCGAGTCGTAATAA
AtLPT4 forward	GCTTTCGCTTTGAGGTTCTTC
AtLPT4 reverse	CCACTGTGCCACATGTTATTG
AtLPT6 forward	TGTGCCTGGTTCTTGCTTTA
AtLPT6 reverse	GGGAGATCGACACCACATTTAG
AtPER36 forward	GAACCATGGAAAGCGAGAAAC
AtPER36 reverse	CTCTAGCAACGAGAGCCAATAG
AtAP2 forward	ATTTGGGACTGTGGGAAACA
AtAP2 reverse	CGCTTCTACTCCACGGAATTTA
AtMUM1 forward	CAAGGTAAAGCGAAGGAGCA
AtMUM1 reverse	CCGCCAAGAGAAGGATGATTAG
AtGL2 forward	TCTCTATCTCTCGCTGGGATATT
AtGL2 reverse	GGTGGTGTGACGATGATACTT
AtTT2 forward	TGGTTCCTGCTCAAGGTAATC
AtTT2 reverse	AGGCTCAACAAGTGAAGTCTC
AtTT8 forward	GCAAAGAGCATCAGCAAGTG
AtTT8 reverse	CGCGGTAGCCTCTTATCTTTAG
AtEGL3 forward	ACAATGGCAACCGGAGAAA
AtEGL3 reverse	AGCAGAGACAGACCAGAAGA
AtTTG1 forward	CTCTCCTTCGAGCATCCTTATC
AtTTG1 reverse	TCCCAAAGACGGAGGAAATC
AtTTG2 forward	CTGGAGGAAATACGGACAGAAG
AtTTG2 reverse	CTTGACCTTCCACTGATCTCTC
AtMyB61 forward	CTTCACTCTGCTCGATGATTCC
AtMyB61 reverse	GGAAGACTAATCGAGGGCTTTAC

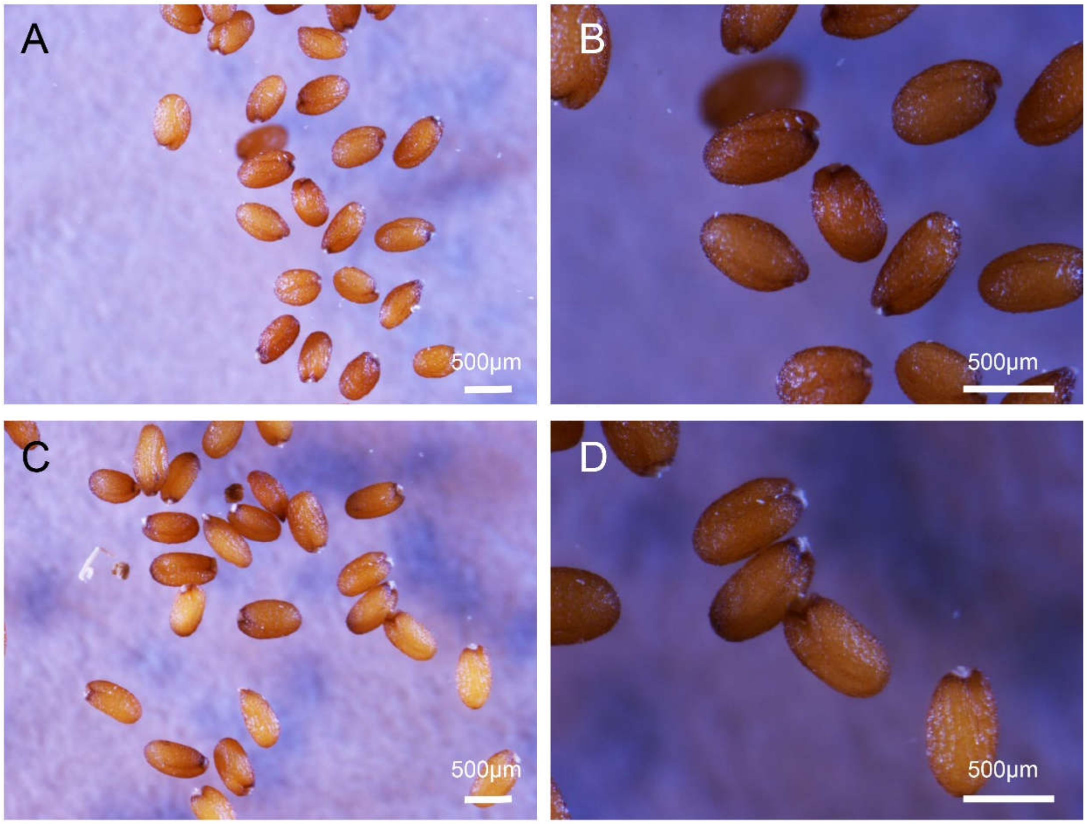

Figure S1. Appearance of WT and *Ataae3* mature dry seeds. Photos of WT (**A** and **B**) and *Ataae3* (**C** and **D**) seeds were taken under light microscopy. The seeds appeared indistinguishable from each other. Scale bars=500µm.
